# Pedicled anterolateral thigh flap: A versatile flap for complex regional defect reconstruction

**DOI:** 10.3205/iprs000174

**Published:** 2023-07-25

**Authors:** Jiten Kumar Mishra, Shamendra Anand Sahu, Moumita De, Aparajita Saha

**Affiliations:** 1 Department of Burns & Plastic Surgery, All India Institute of Medical Sciences, Raipur, India

**Keywords:** pedicled ALT flap, ALT flap, perineum, thigh, Fournier’s gangrene

## Abstract

**Objectives:**

Soft-tissue defects of the lower abdomen, perineum, groin, and trochanteric area often involve the loss of composite tissue components and are technically challenging to reconstruct. The goals of reconstruction should include the replacement of the defect with a suitable soft-tissue flap that provides stable coverage while protecting important exposed structures. However, there are limited locations in this region for the creation of pedicled flaps for complex defect reconstruction. The pedicled anterolateral thigh (ALT) flap is considered superior to other comparable flaps due to its varying soft-tissue components and long pedicle with consistent anatomy that allow the reconstruction of locations that are difficult to reach without significant flap donor site morbidity. Herein, we present a case series of our experience of using a pedicled ALT flap to reconstruct regional defects over a range of locations.

**Methods:**

The present study comprised ten patients who underwent surgical reconstruction of soft-tissue defects of the lower abdomen, groin, trochanteric, scrotal, and penoscrotal defects using a pedicled ALT flap over a two-year period. The flap was customized according to the defect when required.

**Results:**

In our case series, flap loss was not observed with only a few minor complications. All patients accepted the aesthetic appearance of the flap recipient site area without requesting revision surgery. The donor site was closed primarily in half of all cases, with split skin grafting applied in the remaining patients. Graft take at the flap donor site was satisfactory in all cases.

**Conclusion:**

A pedicled ALT flap is a reliable and suitable option for complex soft-tissue reconstruction for regional soft-tissue defects of the lower abdomen and perineum.

## Zusammenfassung

**Zielsetzung:**

Weichteildefekte des Unterbauchs, des Dammes, der Leiste und des Trochanterbereichs gehen häufig mit dem Verlust von Verbundgewebekomponenten einher und sind technisch schwierig zu rekonstruieren. Zu den Zielen der Rekonstruktion sollte der Ersatz des Defekts durch einen geeigneten Weichgewebelappen gehören, der eine stabile Deckung bietet und gleichzeitig wichtige freiliegende Strukturen schützt. In dieser Region gibt es jedoch nur wenige Stellen, an denen gestielte Lappen für die Rekonstruktion komplexer Defekte angelegt werden können. Der gestielte anterolaterale Oberschenkellappen (ALT) gilt aufgrund seiner unterschiedlichen Weichteilkomponenten und seines langen Stiels mit konsistenter Anatomie als anderen vergleichbaren Lappen überlegen und ermöglicht die Rekonstruktion von schwer zugänglichen Stellen ohne signifikante Morbidität der Lappenspenderstelle. Wir stellen hier eine Fallserie unserer Erfahrungen mit der Verwendung eines gestielten ALT-Lappens zur Rekonstruktion regionaler Defekte an verschiedenen Stellen vor.

**Methoden:**

Die vorliegende Studie umfasste zehn Patienten, die sich über einen Zeitraum von zwei Jahren einer chirurgischen Rekonstruktion von Weichteildefekten des Unterbauches, der Leiste, des Trochanter, des Skrotums und des Penoskrotums mit einem gestielten ALT-Lappen unterzogen. Der Lappen wurde bei Bedarf an den jeweiligen Defekt angepasst.

**Ergebnisse:**

In unserer Fallserie wurde kein Lappenverlust beobachtet und es traten nur wenige kleinere Komplikationen auf. Alle Patienten akzeptierten das ästhetische Erscheinungsbild der Empfängerregion des Lappens, ohne eine Revisionsoperation zu verlangen. In der Hälfte aller Fälle wurde die Entnahmestelle primär verschlossen; bei den übrigen Patienten wurde eine Spalthauttransplantation durchgeführt. Die Transplantataufnahme an der Lappenentnahmestelle war in allen Fällen zufriedenstellend.

**Schlussfolgerung:**

Ein gestielter ALT-Lappen ist eine zuverlässige und geeignete Option für komplexe Weichteilrekonstruktionen bei regionalen Weichteildefekten des Unterbauches und des Perineums.

## Introduction

Microvascular free tissue transfer allows the reconstruction of complex defects. However, regional pedicle flaps are still used for reconstructive surgery as they avoid the need for microsurgical procedures and the associated risk of anastomotic failure. Although the anterolateral thigh (ALT) flap is considered a workhorse flap for free tissue transfer, the pedicled ALT flap has similar benefits to the free anterolateral thigh flap in terms of reliable vascularity, versatile soft-tissue component, adequate pedicle length, large flap size, and acceptable donor site morbidity [1], [2]. Here, we present a case series describing the successful use of the pedicled ALT flap for the reconstruction of regional defects with no major intraoperative or postoperative complications.

## Patients and methods

The present case series comprises ten patients that underwent surgical reconstruction for various regional defects using a pedicled ALT flap between 2019 and 2022, including nine male patients and one female patient. Each case required an interdisciplinary team approach. The plastic surgery team performed reconstruction with a pedicled ALT flap after the primary pathology had been managed by a different surgical team with the creation of a soft-tissue defect. Soft-tissue defects were in the mid to lower abdomen, perineum, groin, or trochanteric regions. Patients were followed-up at regular intervals with a minimum follow-up period of one month to a maximum of two and half years. Study data were obtained from case records, follow-up records, and preoperative as well as postoperative photographs (Table 1 (Tab. 1)).

### Technical considerations

The procedure for harvesting a pedicled ALT flap shares common steps with harvesting a free ALT. However, some intraoperative technical considerations are required, which were adopted in the present case series.

- A flap skin paddle should be more distally placed, as required. Preoperative and intraoperative localization of the distal perforator can aid in the design of an eccentric paddle to increase the pedicle length and arc of rotation.
- Other side branches of the descending lateral circumflex femoral artery can be clipped to increase the pedicle length.
- A small soft-tissue fringe around the pedicle can be preserved to prevent kinking and spasm of the vasculature.
- An acute turn of the pedicle can be avoided by routing the pedicle below the rectus femoris or sartorius muscles while reconstructing abdominal, groin, or perineal defects. Tunneling the flap under the rectus femoris muscle increases the reach of the flap proximally by approximately 2 cm [3].

Adequate subcutaneous tissue tunnels must be created to accommodate the pedicle without compression. In cases with a bulkier flap where a sizable subcutaneous tunnel is required to deliver the flap to the recipient site or when surrounding scar tissue prevents wide undermining, the tunnel opened to accommodate the pedicle and the overlying skin was closed primarily.

## Results

All flaps survived completely in the present series. The flap size ranged from 80 cm^2^ to 320 cm^2^, with a mean flap size of 140 cm^2^. The pedicle length ranged from 8 cm to 13 cm. Septocutaneous perforators were identified in 20% and musculocutaneous in 80% of all cases. Dehiscence at the insertion margin requiring resuturing was done in two cases. None of the patients requested debulking of the reconstructed flap. In half of all cases, the flap donor site was closed primarily, with split skin grafting performed in the remaining cases. Loss of the split skin graft at the flap donor site which required regrafting was observed in one case. Functional morbidity of the donor site was not observed in this case series. Seroma formation and lymphatic discharge around the suture line lasting for two to three weeks were observed in cases where defects from inguinal node block dissection were reconstructed with a pedicled ALT flap.

### Illustrative cases

#### Case 1

**Figure 1:**
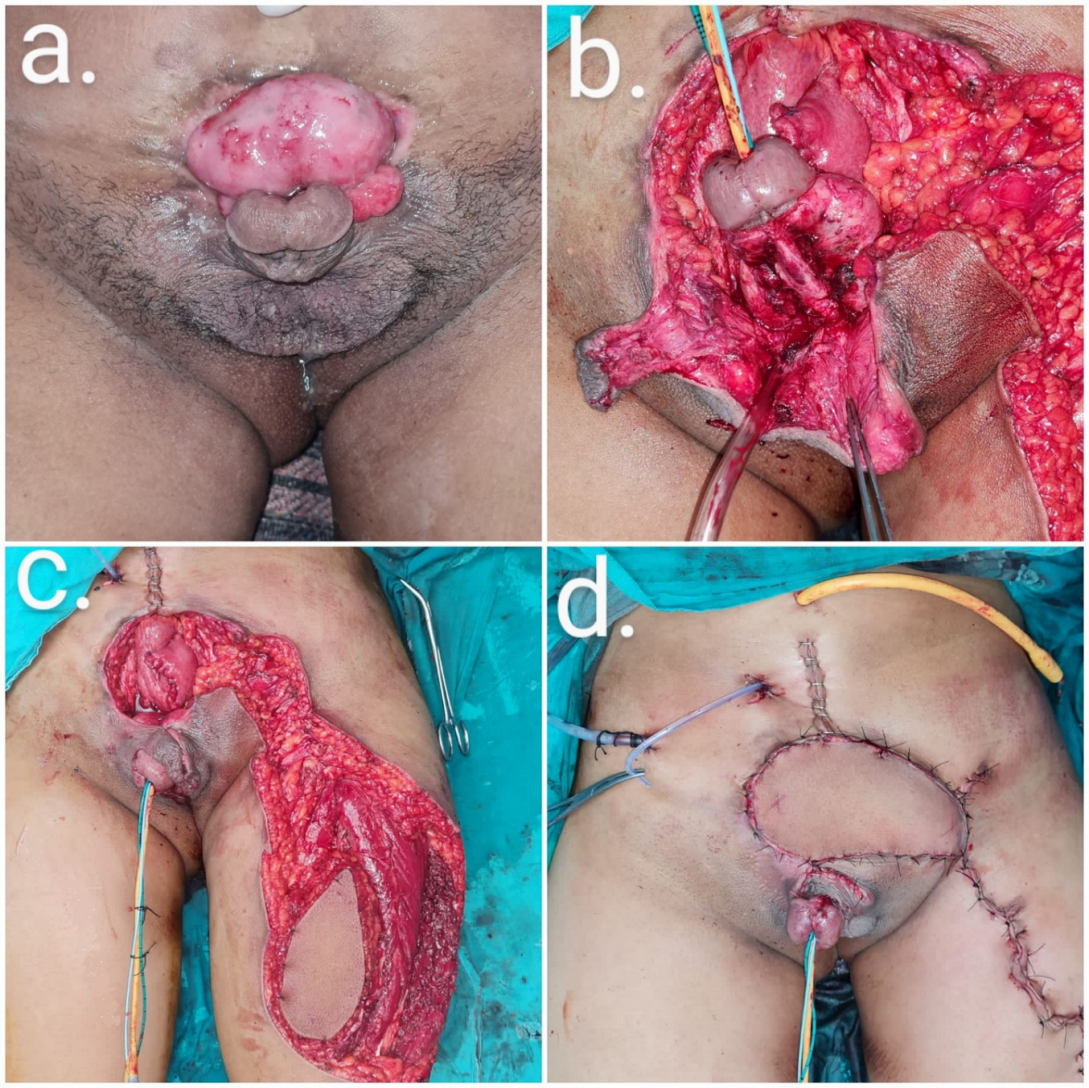
Exstrophy-epispadias complex reconstruction (case 1)

A 22-year-old male patient presented with an exstrophy-epispadias complex. The urology team performed ileocystoplasty and epispadia repair. After neobladder creation and bladder neck repair, the abdominal defect was reconstructed with a pedicled ALT flap from the left thigh. Urosurgeons created a stoma in the right iliac fossa for intermittent self-catheterization. During harvesting of the ALT flap, a muscle cuff was included near the perforators, and the fascia lata was taken with the flap. The abdominal fascial defect was reconstructed with the fascia lata component. Postoperatively, the flap survived well but dehiscence developed at the inferior margin of insetting, which healed by secondary intention. There were no complaints of abdominal herniation during the follow-up period (Figure 1 (Fig. 1)).

#### Case 2

**Figure 2:**
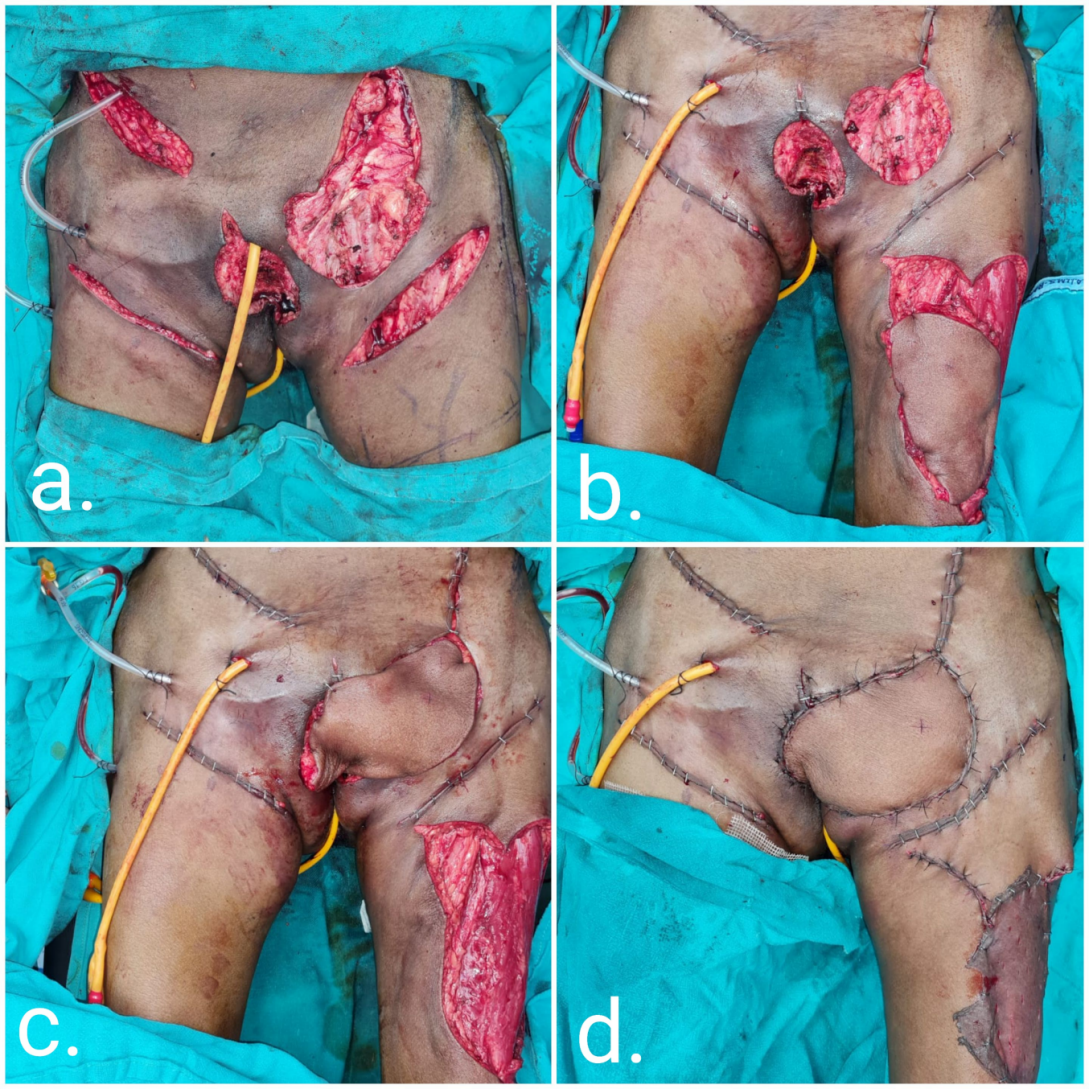
Total penectomy with left inguinal node dissection defect reconstruction (case 2)

Total penectomy with bilateral node dissection and perineal urethrostomy was performed in a case of recurrent carcinoma of the penis with inguinal nodal metastasis that was fixed to the skin on the left side. After penectomy and local excision of the tumor, the soft-tissue defect was extended from the left inguinal region to the pubic area with exposure of the femoral vessels. The defect was reconstructed with a pedicled ALT flap from the left thigh. Lymphatic oozing from the groin area was observed postoperatively, which resolved spontaneously. Dehiscence developed at the opposite inset margin, which was managed conservatively (Figure 2 (Fig. 2)).

#### Case 3

**Figure 3:**
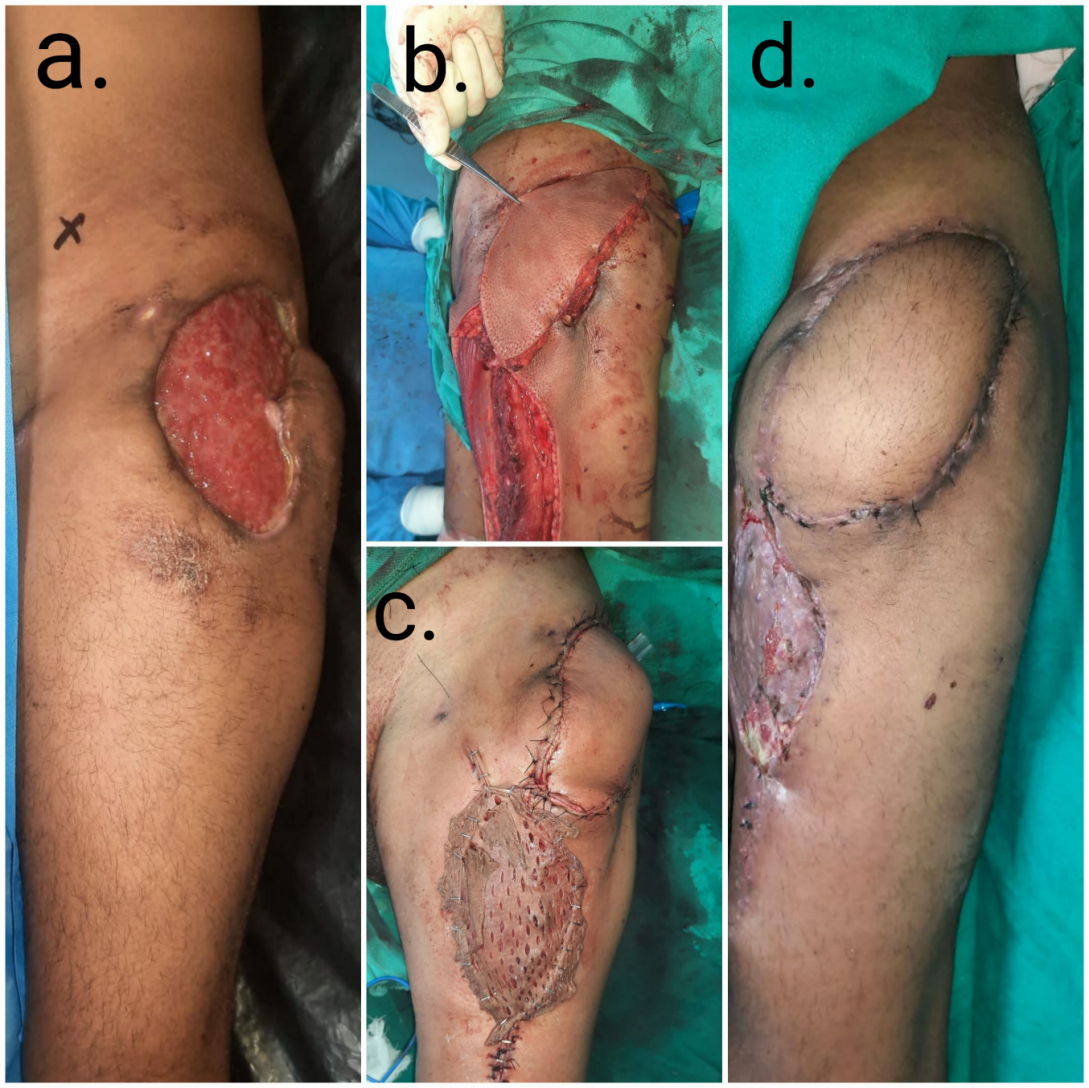
Left trochanteric soft tissue defect reconstruction (case 3)

A young patient presented with a chronic wound over the left greater trochanteric area following injection site cellulitis and soft-tissue necrosis. The wound did not heal after split skin grafting performed at an outside hospital. A pedicled ALT flap was performed for coverage of the wound. The flap donor area was closed partially, and the rest of the area was skin grafted. The flap reconstruction site healed satisfactorily without recurrent ulceration (Figure 3 (Fig. 3)).

#### Case 4

**Figure 4:**
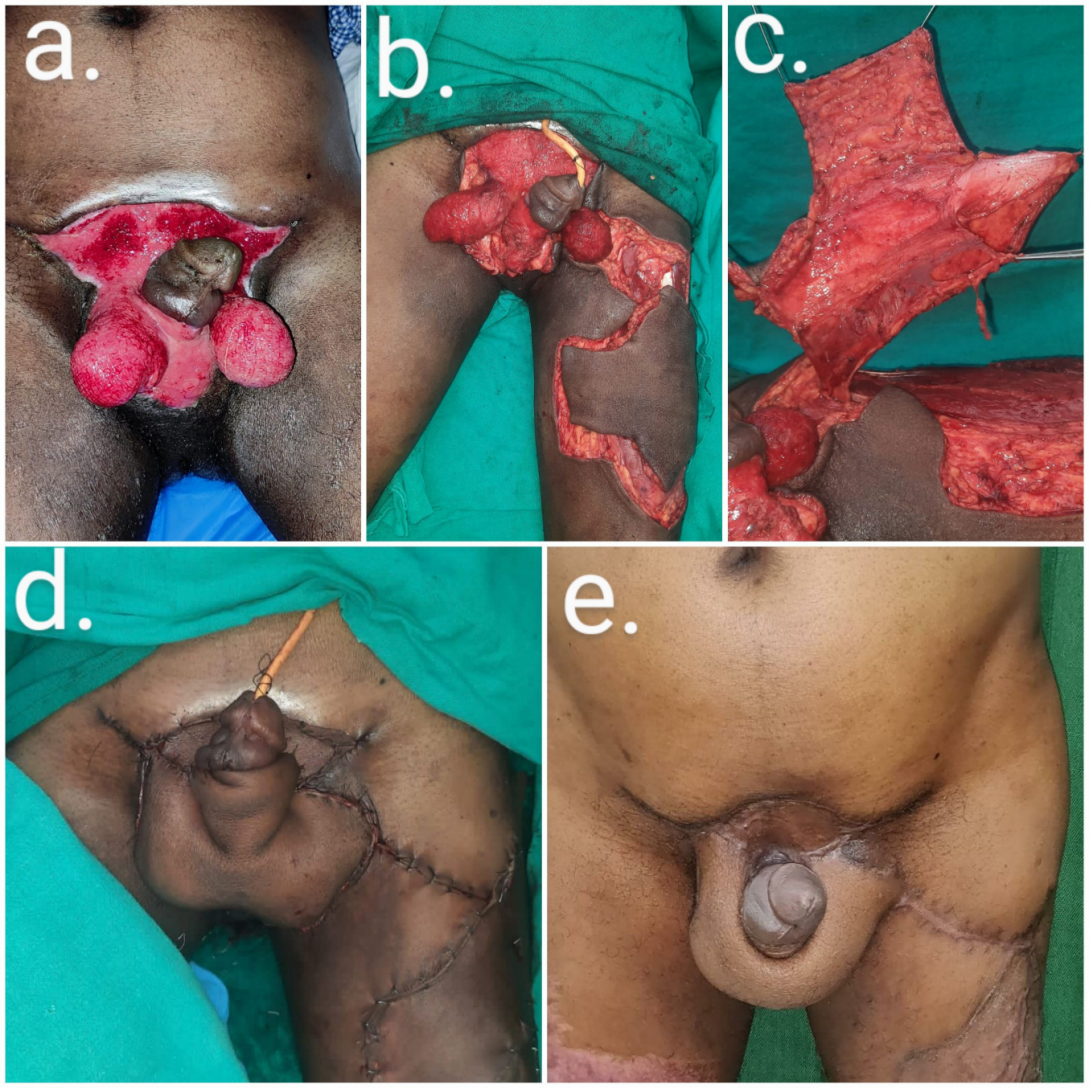
Peno-scrotal defect (post-Fournier’s gangrene) reconstruction (case 4)

A 47-year-old male was diagnosed with Fournier’s gangrene with complete loss of the skin and soft tissue of the scrotum, penis, and supra-pubic area. We reconstructed the soft tissue of the penile shaft and formed a neoscrotum using a customized ALT flap. A conventional ALT flap with a flag-shaped projection was designed to cover the scrotum and penis. The suprafascial flap was elevated to provide a thin, pliable coverage that mimicked the natural penile shaft (Figure 4 (Fig. 4)).

#### Case 5

**Figure 5:**
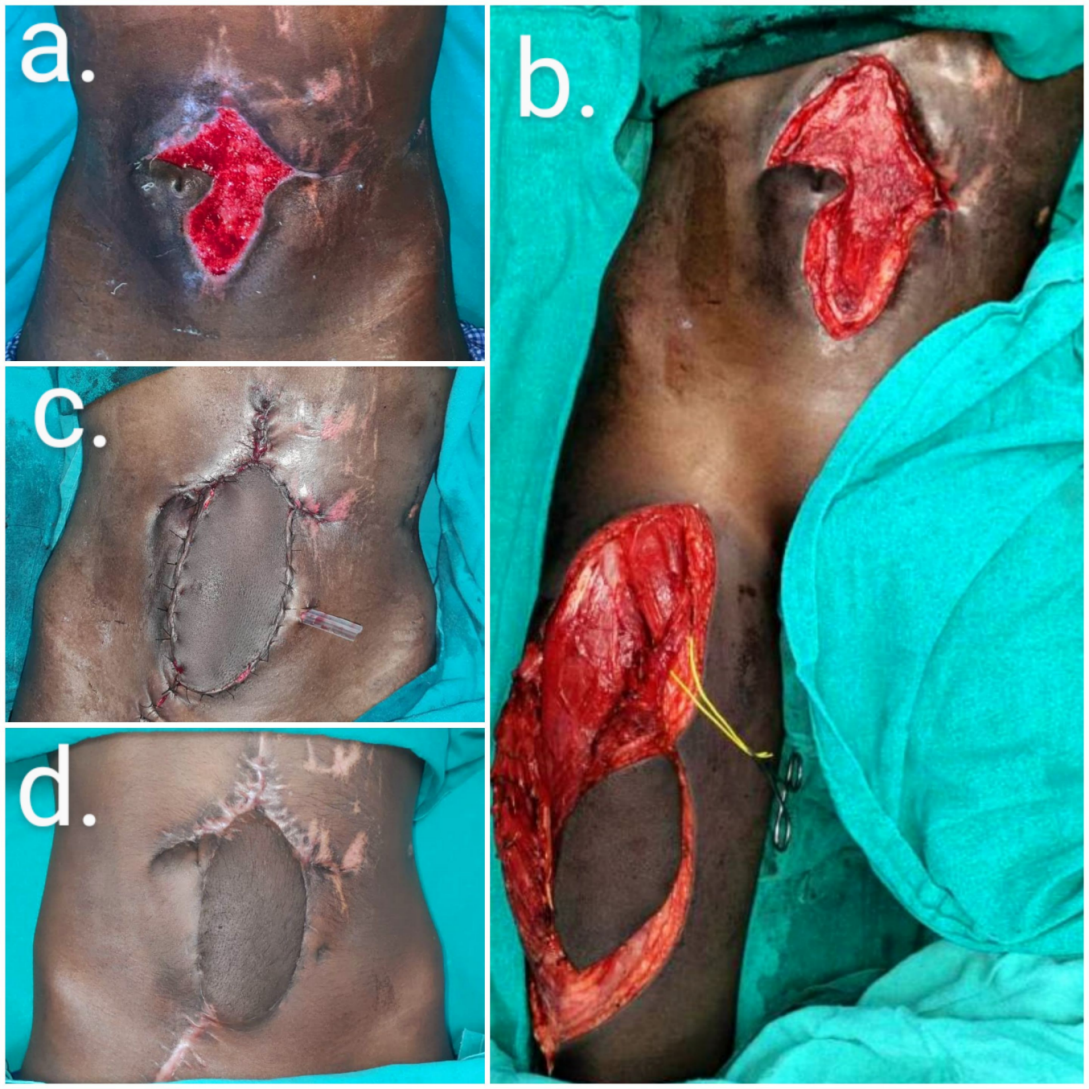
Abdominal defect reconstruction (case 5)

A 16-year-old male patient with penetrating abdominal trauma with associated bowel injury underwent resection anastomosis of the small bowel. Postoperatively, abdominal wound dehiscence developed. He was referred to us with a defect over the mid abdominal region. During planning of the ALT flap, a distal perforator was mapped and an eccentric skin paddle was designed based on the distal perforator to gain pedicle length. Conventionally, the skin paddle is designed centered on a 3 cm circle as the midpoint between the anterior superior iliac spine and the superolateral border of the patella. The fascia lata was harvested with the flap and used to repair the rectus defects. The flap donor site was closed primarily. Minor dehiscence developed at the insertion that required resuturing, with an acceptable contour observed at follow-up (Figure 5 (Fig. 5)).

## Discussion

**Figure 6:**
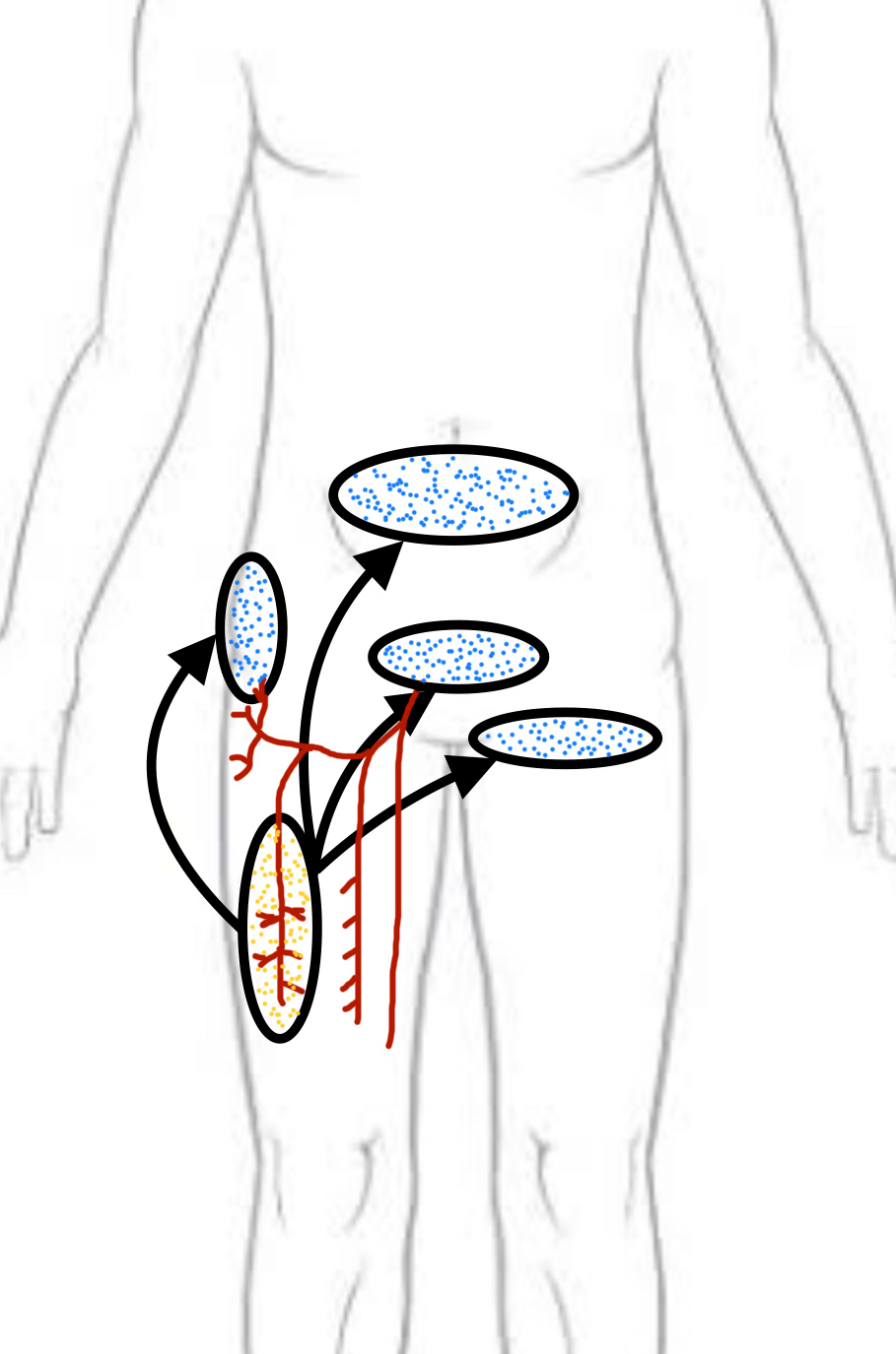
Figure depicting reach and arc rotation of pedicled anterolateral thigh flap

After the free ALT flap was first described by Song et al. in 1984, the ALT flap has become a widely used tool for the reconstruction of a range of soft-tissue defects affecting the head, neck, and extremities due to its favorable characteristics [4]. The use of the pedicled ALT flap for regional defect reconstruction was first described by Kimata et al. in three cases of abdominal defect reconstruction [5]. The pedicled ALT flap can reach a wide range of regional locations, including the mid-to-lower abdomen, ipsilateral groin, perineum, trochanteric area, and the contralateral groin with a long pedicle length (Figure 6 (Fig. 6)). The maximal pedicle length that can be achieved with the ALT flap is approximately 37 cm (descending branch of lateral circumflex femoral artery) when used to cover contralateral groin defects [6], [7]. Abdominal defects often do not allow reconstructive surgeons to borrow soft tissue from the abdomen by component separation or raising local flaps due to the presence of stomas, trauma, congenital defects, or previous oncological resection. Of the three cases requiring abdominal defect reconstruction in the present series, two cases had adult exstrophy-epispadias complex. Lower abdominal soft-tissue defects of the adult exstrophy-epispadias complex require the replacement of both fascial defects and soft-tissue. In our third case with an abdominal wall defect due to penetrating abdominal injury, secondary reconstruction of the mid abdominal defect was performed with a pedicled ALT flap. In this case, the branch to the rectus femoris and the transverse branch to the tensor fascia lata were clipped to gain a pedicle length of 13 cm, with the distal limit of the flap reaching 4 cm above the umbilicus. Kimata et al. described the reconstruction of abdominal defects reaching 8 cm above the umbilicus [4]. For abdominal, groin, and perineal defects, the flap pedicle can be routed below the rectus and sartorius muscles [8]. Though technically challenging, the pedicled ALT flap can also be tunneled intermuscularly to reconstruct complex posterior thigh defects [9]. The ALT flap provides a stable and homogenous bulk as required by the abdominal defect. To reconstruct these defects, a segment of the vastus lateralis muscle and fascia lata can be incorporated with the flap without intramuscular dissection of the perforators. The pedicled ALT flap can also be used with prosthetic mesh to cover abdominal defects with exposed intestines [10]. Although the vertical rectus abdominis is a reliable flap, this was not an option in any of our cases of abdominal defects due to the questionable integrity of the pedicle [11]. 

For reconstruction of areas where identifying suitable recipient vessels may be difficult, such as cases with tissues defects around the trochanter area, the pedicled ALT flap provides highly vascular soft-tissue coverage that facilitates wound healing. Trochanteric defects are usually reconstructed with a tensor fascia lata (TFL) flap. However, the TFL flap has limitations, including variable distal vascularity and dog ear deformities at the recipient site, which often require further corrective surgery [12]. The pedicled ALT flap can provide durable and reliable tissue for soft-tissue coverage of trochanteric pressure sores [13].

Groin defect reconstruction is indicated for exposed femoral vessels, which is common after inguinal block dissection due to nodal metastasis with overlying skin involvement. Regional techniques, such as the sartorius switch or the use of a vertical rectus abdominis flap, are commonly used. The sartorius switch provides barely sufficient coverage of exposed vessels, which may be inadequate in cases requiring postoperative radiotherapy or with large skin and soft-tissue defects. Although the pedicled vertical rectus abdominis flap provides skin and muscle components, the loss of abdominal muscle may increase the risk of hernia formation or result in laxity of the abdominal wall [14].

Defects resulting from Fournier’s gangrene are technically challenging to reconstruct. The goal is to reconstruct a neoscrotum while providing stable and vascularized coverage of the exposed testes to reduce the risk of infection. The most commonly used technique for covering the penoscrotal region is split skin grafting. Reported complications of split skin grafting are repeated graft loss, scarring, and a shortening of the penile length due to scar contracture leading to painful erections [15]. Alternative techniques, such as the medial thigh flap, gracillis flap, Singapore flap, and inferior gluteal flap, have shortcomings that include graft loss, flap loss, inadequate flap size, and the unnatural look of the neoscrotum [3], [16], [17]. The use of a pedicled ALT flap provides a one-stop solution that provides coverage of both testes, even in cases with loss of the entire scrotal skin. In our case of Fournier’s gangrene with penoscrotal involvement (case 4), we customized the ALT flap by elevating a thin suprafascial flap to mimic the penoscrotal skin. In addition to the elliptical design of the flap that allowed creation of the neoscrotum, a flag-shaped medial unit for penile coverage was also created (Figure 4 (Fig. 4)). Using this design for penoscrotal defect reconstruction, both the penile shaft and testes were covered with pliable soft tissue. At a follow-up visit, the patient reported the resumption of sexual activity two months after surgery without painful erections or a decrease in penile length, and the aesthetic outcomes in terms of skin pliability and quality, bulk, and shape of the scrotum are acceptable to the patient. Sapino et al. reported a similar case with a scrotal perineal defect where a rectangular extension on the posterior margin was created to cover the perineal region, which the authors termed the Sombrero shape flap [18]. The ALT flap is well suited to reconstruction of the scrotum due to shrinkage of the bulk of the flap over time. The common modality of reconstructing soft-tissue loss from the penile shaft using split-thickness skin grafting is associated with complications that include graft loss, scarring, and contracture of the penile shaft, thereby leading to painful erections. Thus, the ALT flap can be customized to simultaneously reconstruct the penis and scrotum in patients with penoscrotal defects due to infective or traumatic tissue loss.

A pedicled ALT flap is technically easy to create with less operative time as microvessel preparation and anastomosis are not required. In the four cases that had undergone surgery performed by our urology team (two cases of exstrophy-epispadias and two cases of inguinal block dissection defects due to metastasis from carcinoma of the penis) reconstruction was undertaken simultaneously with a pedicled ALT flap where the assisting staff were not technically versed in complex microvascular reconstruction procedures. Further, these patients required less rigorous postoperative flap monitoring.

The main shortcomings of the pedicled ALT flap are the limited reach of the flap beyond the arc of rotation, thereby preventing the use of this technique for the repair of defects in distant regions, such as the upper abdomen, and the lack of freedom when orienting the skin paddle, which is possible with the use of a free ALT. Using the design of skin flap paddles and incorporating techniques to increase pedicle length, the versatility of the pedicled ALT flap is considered superior to other regional flap options when reconstructing complex regional soft-tissue defects.

## Conclusion

Use of the pedicled ALT flap avoids the need for free tissue transfer when reconstructing regional defects. The pedicled ALT flap allows the swift reconstruction of large and complex soft-tissue defects, and therefore represents a suitable alternative to other commonly used techniques. Accordingly, the pedicled ALT flap should be considered the first choice method for regional defect reconstruction. This technique is particularly customizable due to the versatile and expendable tissue components of the ALT flap.

## Notes

### Competing interests

The authors declare that they have no competing interests.

## Figures and Tables

**Table 1:**
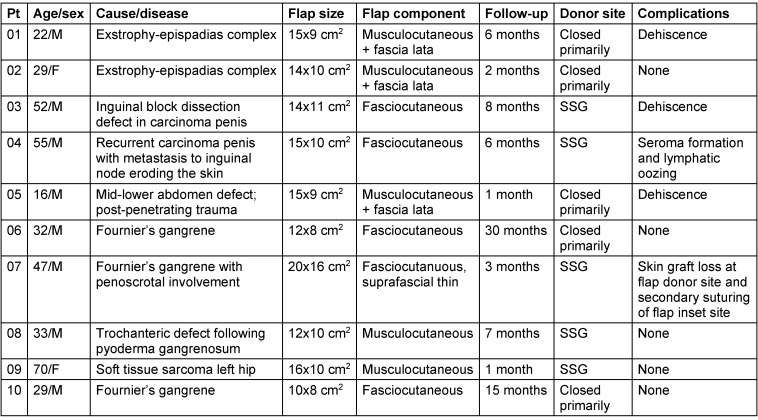
Demography of the patients, procedure, and the outcome
